# Artificial Intelligence and Colposcopy: Automatic Identification of Cervical Squamous Cell Carcinoma Precursors

**DOI:** 10.3390/jcm13103003

**Published:** 2024-05-20

**Authors:** Miguel Mascarenhas, Inês Alencoão, Maria João Carinhas, Miguel Martins, Pedro Cardoso, Francisco Mendes, Joana Fernandes, João Ferreira, Guilherme Macedo, Rosa Zulmira Macedo

**Affiliations:** 1 Precision Medicine Unit, Department of Gastroenterology, São João University Hospital, Alameda Professor Hernâni Monteiro, 4200-427 Porto, Portugal; miguel.pedro96@gmail.com (M.M.); pedromarilio@gmail.com (P.C.); guilhermemacedo59@gmail.com (G.M.); 2 Faculty of Medicine, University of Porto, Alameda Professor Hernâni Monteiro, 4200-427 Porto, Portugal; 3 Department of Gynecology, Centro Materno-Infantil do Norte Dr. Albino Aroso (CMIN), Santo António University Hospital, Largo da Maternidade Júlio Dinis, 4050-061 Porto, Portugal; inesalencoao@gmail.com (I.A.); mjcarinhas20@gmail.com (M.J.C.); rosazulmira@gmail.com (R.Z.M.); 4 Faculty of Engineering, University of Porto, Rua Dr. Roberto Frias, 4200-065 Porto, Portugal; joana.fernandes@digestaid.health (J.F.); joao.ferreira@digestaid.health (J.F.)

**Keywords:** cervical squamous cell carcinoma, LSIL, HSIL, colposcopy, artificial intelligence

## Abstract

**Background/Objectives**: Proficient colposcopy is crucial for the adequate management of cervical cancer precursor lesions; nonetheless its limitations may impact its cost-effectiveness. The development of artificial intelligence models is experiencing an exponential growth, particularly in image-based specialties. The aim of this study is to develop and validate a Convolutional Neural Network (CNN) for the automatic differentiation of high-grade (HSIL) from low-grade dysplasia (LSIL) in colposcopy. **Methods**: A unicentric retrospective study was conducted based on 70 colposcopy exams, comprising a total of 22,693 frames. Among these, 8729 were categorized as HSIL based on histopathology. The total dataset was divided into a training (90%, *n* = 20,423) and a testing set (10%, *n* = 2270), the latter being used to evaluate the model’s performance. The main outcome measures included sensitivity, specificity, accuracy, positive predictive value (PPV), negative predictive value (NPV), and the area under the receiving operating curve (AUC-ROC). **Results**: The sensitivity was 99.7% and the specificity was 98.6%. The PPV and NPV were 97.8% and 99.8%, respectively. The overall accuracy was 99.0%. The AUC-ROC was 0.98. The CNN processed 112 frames per second. **Conclusions**: We developed a CNN capable of differentiating cervical cancer precursors in colposcopy frames. The high levels of accuracy for the differentiation of HSIL from LSIL may improve the diagnostic yield of this exam

## 1. Introduction

Cervical cancer poses a significant global challenge, with recent estimates indicating an incidence of 13.3 cases per 100,000 women-years [1]. This burden is particularly pronounced in low-income countries, where both incidence and mortality rates are higher [2]. Central to this disease’s carcinogenesis is the role played by Human of Papillomavirus (HPV) infection, which affects over 80% of sexually active individuals at some point in their lives [3]. While most HPV infections are temporary, the persistence of the infection, especially in high-risk oncogenic types, significantly contributes to the development of precursor dysplastic lesions, including both low-grade and high-grade squamous Intraepithelial Lesions (LSILs and HSILs, respectively) [4].

Distinguishing between LSILs, characterized by mild dysplasia, and HSILs, characterized by moderate/severe dysplasia and a higher probability of progressing to invasive malignancy, is critical for guiding subsequent medical treatments [5]. While LSILs may resolve spontaneously due to a decreased propensity for progression, HSILs are considered to be an actual precancerous lesion and require prompt treatment, often using ablative techniques or excisional procedures [6].

Despite the discouraging prognosis of advanced disease stages, early detection facilitates the effective management of cervical cancer [7]. The 90-70-90 targets emphasize the importance of adequate vaccination rates, HPV screening, and the early treatment of pre-invasive disease [8]. Screening typically involves HPV testing and/or cytological examination, with colposcopy is recommended in cases of altered results [9]. In fact, colposcopy is considered the gold standard for diagnosing cervical cancer, due to its magnification capabilities, allowing improved morphological mucosal characterization, as well as the ability to perform targeted biopsies and treat suspected lesions [10]. In this scenario, the primary aim of colposcopy in cervical cancer screening is to detect and treat HSILs before they advance to cervical cancer.

Staining methods can be added at the time of colposcopy, to increase its diagnostic yield (HSILs are expected to appear whitish in acetic acid application and show no coloration with Lugol’s iodine) [11]. While prospective studies support the sequential application of acetic acid and Lugol’s staining, increasing the diagnostic performance of colposcopic procedures essentially through heightened sensitivity, there are inherent limitations to this approach [12]. Suboptimal specificity and interobserver variability contribute to a notable number of false positives, even when incorporating staining in colposcopy. This exam continues to depend heavily on the clinician’s skill and has high intra and interobserver variability. The identification of HSILs is still suboptimal, with a recent study demonstrating an accuracy of approximately 70% for the identification of this lesion type [13].

Challenges in achieving optimal accuracy persist, emphasizing the need for ongoing refinement in order to enhance the overall reliability of cervical cancer detection during colposcopy. In this context, artificial intelligence (AI) models could have a role in increasing colposcopy’s cost-effectiveness, leading to more target treated decisions and potentially reducing unnecessary procedures. Convolutional Neural Networks (CNNs) are deep learning AI models inspired by the human visual cortex, specifically designed for image patterns analysis [14]. The advantages of CNN development have been explored across several medical areas [15,16].

The aim of this study is to develop and validate a Convolutional Neural Network (CNN) for the automatic differentiation of HSILs from LSILs in colposcopy, using still frames of all the phases of colposcopy exam (non-stained, acetic acid, Lugol’s iodine, and post-manipulation).

## 2. Materials and Methods

We retrospectively collected colposcopies performed between December 2022 and January 2023 in Centro Materno Infantil do Norte, in Porto, Portugal. The colposcopic procedures were captured using a Zeiss 150 FC colposcope. Subsequently, the collected videos were segmented into still frames using a VLC Media Player (VideoLan, Paris, France). The frames underwent a comprehensive review, resulting in the inclusion of a total of 22,693 frames in the dataset.

This study follows a non-interventional paradigm, given the retrospective nature of collection of previously performed colposcopies. Furthermore, the study did not precipitate modifications to the therapeutic conduct in any instance. Ethical committee approval was obtained prior to the start of the study (IRB 2023.157(131-DEFI/123-CE)), adhering to the principles outlined in the Helsinki declaration.

Colposcopies were performed by one expert (M.J.C.) and all the collected procedures followed the current standards of practice. This includes the proper execution of the biopsies of suspected lesions and the preservation of sampled tissue. Each procedure may be divided into four segments, as follows: initial non-stained observation, 3% acetic acid solution observation, Lugol observation, and after therapeutic manipulation (e.g., after laser ablation, plasma coagulation, or surgical ablation). While not all colposcopy examinations included all four segments (e.g., some may lack Lugol’s staining or adequate post-procedure images), the dataset includes frames from these four categories (frames without staining, frames following the application of acetic acid, frames following the application of Lugol’s solution, and post-manipulation frames). This means that individual colposcopy procedures within the dataset may include any combination of these categories.

**Figure 1:**
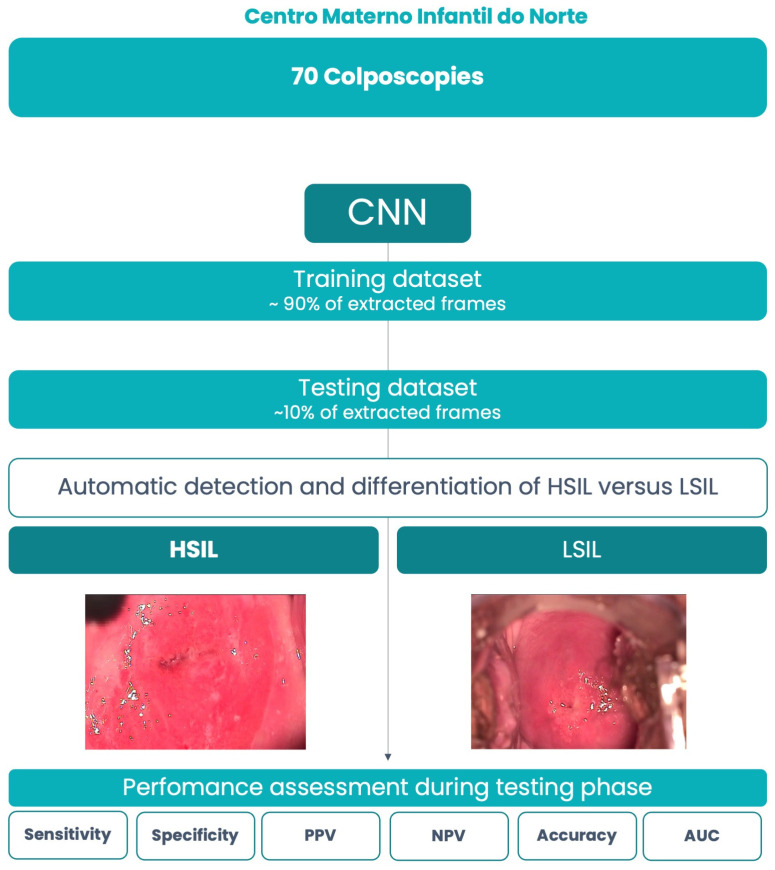
Flowchart describing the study design. CNN: Convolutional Neural Network; HSIL: high-grade squamous intraepithelial lesion; LSIL: low-grade squamous intraepithelial lesion; PPV: positive predictive value; NPV: negative predictive value; AUC: area under the curve.

Among the total dataset (*n* = 22,693), 8729 frames were classified as HSILs, while the remaining 13,964 frames were categorized as LSILs. The histological report from the biopsy taken during the colposcopic examination of the observed lesions was consistently consulted, in order to classify each corresponding colposcopy frame as LSIL or HSIL (histological assessment is the ground truth of this CNN model). The complete dataset was split into two parts—a training set comprising 20,423 frames (90% of the total dataset) and a testing set with 2270 frames (10% of the total dataset). Each frame was exclusively assigned to either the training or testing category. We used a stratified sampling strategy, assuring that the distribution of lesions remained consistent across data splits. The testing set served as an independent validation for the CNN. Figure 1 depicts the study flowchart.

We constructed this CNN using a ResNet model. The model’s weights were pre-trained using ImageNet, a comprehensive image dataset designed for object recognition. We kept the initial convolutional layers to transfer its learned features to our model. The last fully connected layers were removed and new fully connected layers were attached, based on the number of classes needed for classifying colposcopy frames. The model’s architecture includes two blocks, each comprising a fully connected layer followed by a dropout layer with a drop rate of 0.3. Subsequent to these blocks, we incorporated a dense layer, whose size was determined using the binary classification (HSIL or LSIL). The hyperparameters, including learning rate (0.00015), batch size (128), and number of epochs (10), were fine-tuned through a process of trial and error. Data preparation involved using FFMPEG, Pandas, and Pillow libraries, while PyTorch was utilized to run the model. The computational system was driven using a double NVIDIA Quadro RTXTM 80,000 graphic processing unit (NVIDIA Corp, Santa Clara, CA, USA), alongside an Intel 2.1 GHz Xeon Gold 6130 processor (Intel, Santa Clara, CA, USA).

**Figure 2:**
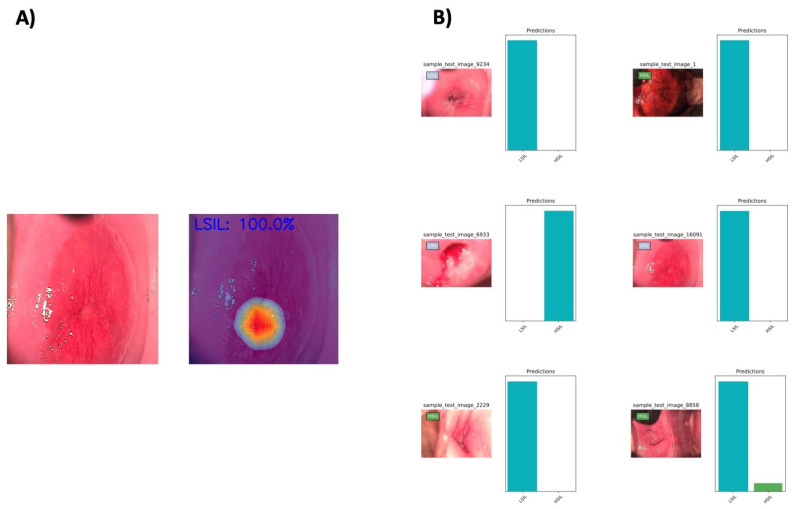
(**A**) Example of generated heatmap showing how the CNN distinguishes a precursor cervical squamous cell carcinoma. (**B**) The graph shows the output obtained using the CNN model. The algorithm’s predicted the probability of being categorized as HSIL or LSIL, as indicated by each bar in the chart. Every frame was assigned to one of these categories, based on the highest probability. The corresponding assessment using histopathological classification, regarded as the gold standard, is indicated in a rectangle in the superior upper corner.

The model calculated the probability of each frame being classified as HSIL or LSIL. Their final classification was determined based on the one category with the highest probability. Subsequently, the CNN’s classification was compared to the corresponding histopathological one, regarded as the gold standard. The primary outcome measures were sensitivity, specificity, accuracy, positive predictive value (PPV), negative predictive valued (NPV), and area under the receiver operating curve (AUC-ROC). Heatmaps were also generated to enhance our understanding of the specific frame regions contributing the most to the CNN’s prediction (Figure 2). The computational performance was assessed by measuring the time required to process all frames in the testing set. Sci-Kit learn was used for statistical analysis [17].

## 3. Results

A combined total of 22,693 frames were used for the development and validation of the CNN, with 8729 frames being classified as HSIL. From the complete dataset, 90% (*n* = 20,423 frames) were allocated for training the algorithm, while the remaining portion was used for independent validation. The confusion matrix of the testing set is presented in Table 1.

**Figure 3:**
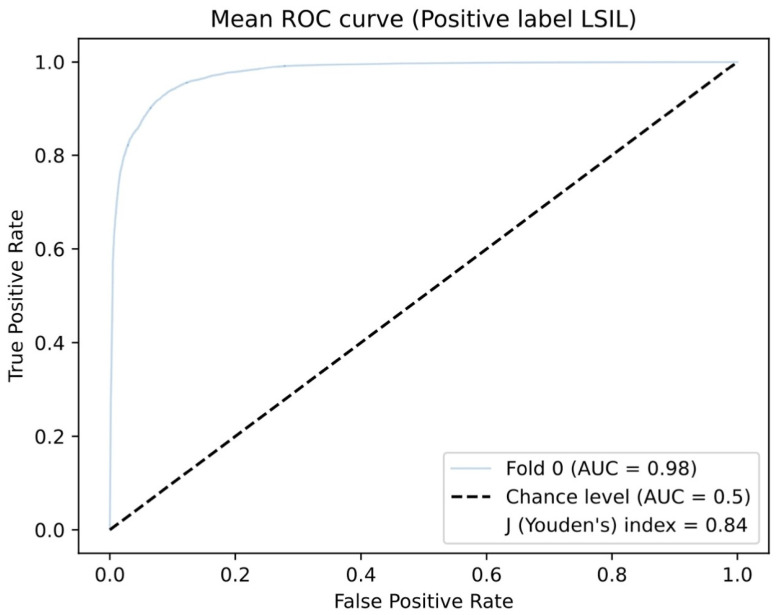
Representative example of an area under the conventional receiver operating characteristic curve (AUC-ROC) of the CNN’s performance in differentiating HSILs from LSILs in colposcopy.

The CNN’s sensitivity was 99.7% and its specificity was 98.6%. The PPV and NPV were 97.8% and 99.8%, respectively. The overall accuracy was 99.0%. The AUC-ROC was 0.98 (Figure 3). The CNN processing time was 112 frames per second.

## 4. Discussion

This study serves as a proof-of-concept deep learning model in gynecology, demonstrating overall good performance metrics in distinguishing between LSILs and HSILs in still cervical frames. Although it is based on retrospective data from a single center, caution should be exercised in interpreting these findings, as their applicability to a broader population may be limited and there is a risk of overfitting the model. Nevertheless, we believe that the development of such deep learning algorithms could propel gynecological endoscopy forward, leading to the improved management and enhancement of women’s health.

Several CNNs have been published to detect and differentiate cervical dysplastic lesions during colposcopy. The first one, published in 2019, used one non-stained frame per procedure across 330 patients, employing a 5-fold cross-validation design [18]. This algorithm achieved 80% sensitivity and 88% specificity, with an overall accuracy of 83% in distinguishing LSILs from HSILs. Subsequently, a large retrospective study involving 22,330 patients, using both non-stained and stained (acetic acid and Lugol) annotated frames, demonstrated a 93% accuracy in detecting dysplastic lesions [19]. Particularly for differentiating HSILs, the CNN exhibited an 85% sensitivity. Another study, with a larger dataset of 19,435 non-stained annotated frames, achieved a 66% sensitivity and a 90% specificity in distinguishing HSILs [20]. A recent publication from 2022 include a study involving 18.006 frames from 6.002 patients, with non-stained and stained ones [21]. The model exhibited an 88% sensitivity and a 94% specificity in differentiating between LSILs and HSILs. Additionally, another study aimed to differentiate normal, LSILs, HSILs, and cervical cancer in non-stained frames, achieving an 86% sensitivity for LSILs and an 82% sensitivity for distinguishing HSILs from other categories [22]. Our results demonstrate that the performance of our algorithm, using a dataset of 22,693 non-annotated frames, was high, with a sensitivity and specificity of 99.7% and 98.6%, respectively, and an accuracy of 99.0% in detecting and differentiating LSILs from HSILs. Due to the insufficient number of histologically confirmed “normal” lesions, it was not possible to conduct a trinary model (non-dysplastic vs. LSILs vs. HSILs) in this study.

There are some strengths of this study that deserve emphasis. Firstly, the model stands outs as the first CNN developed for this clinical problem using a European population. The representability concern is a current topic in the field of data science and AI. The uncertainty arises from questioning if a previously published algorithm would perform effectively in a different population. In addition, it is important to acknowledge that this is the first study to include not only non-stained, but also stained and post-manipulated frames. In a clinical context, this concern is pivotal, as the ultimate goal is to develop a CNN capable of recognizing potential lesions without being influenced by the presence of blood or burn tissue. Moreover, the model development design employed does not necessitate frame annotation for training the CNN, only labeling is required. This can also be an advantage in data development, accelerating the AI algorithm’s pattern analysis when frames lack previous annotations. However, it is crucial to acknowledge that the classification rationale may not always match our initial assumptions. This leads to addressing another study detail, namely the generation of heatmaps. These can be crucial for the explainability of our results, helping in determining whether the algorithm is detecting lesions as anticipated. In this case, it may indicate that the CNN is classifying frames according to our envisioned classification, which can eventually assist in guiding biopsies in the future. It is also worth mentioning that, as expected for this type of AI-enhanced endoscopy algorithm, the model’s development was based on the gold standard of histologic results from biopsies at the specific cervix site, rather than relying on expert opinions, assuring the robustness of the model. Ultimately, our dataset preparation approach consisted in extracting frames not only from different stainings, but also from different cervical locations. Rather than consistently opting for low-ampliation images of the entire cervix, we specifically targeted segments of the cervix displaying a visible lesion (region of interest) that were different from procedure to procedure. This approach enhanced the heterogeneity of anatomical locations represented in the dataset, potentially improving model’s ability to detect lesions in an actual clinical setting.

There are some limitations that should be considered when interpreting these results. Firstly, it is important to note that this is a single-center and retrospective study, impacting the generalizability of the findings. Secondly, although it is simultaneously a strength, given the absence of published research within this specific population, by include a Mediterranean population in our dataset, we may have introduced a demographic bias. Consequently, this also implies that the study’s performance metrics might not be applicable to other populations. Furthermore, the design involved a 90% training and a 10% testing split for the CNN’s development, lacking a procedure split. Consequently, there is a risk of overfitting that cannot be neglected, as similar frames from the same procedure may appear in both training and testing sets, leading to an overestimation of the results. However, the split between training and testing, using a stratified sampling, assured a similar percentage of every lesion type between datasets, assuring a good balance for the training in the detection of each specific lesion. Moreover, it is important to acknowledge that we exclusively used frames from one brand of colposcopes, introducing uncertainty about the CNN’s performance with data from other manufacturers. Additionally, this algorithm was developed only with still frames, implying that its performance may not necessarily reflect its accuracy in real-life video circumstances, although the computational fast performance of 112 frames per second may suggest otherwise.

While we recognize that multiple cervical lesions can occur concurrently in the same colposcopy (e.g., LSIL and HSIL), addressing this complexity would have necessitated a larger dataset and a different research approach. Since our dataset preparation did not entail manual data annotation, and due to the inherent black box nature of these deep learning models, incorporating frames comprising two lesions with different histologies could introduce errors during CNN training. Therefore, although the perspective was to work in future models that could assist physicians in detecting concomitant lesions in real-life scenarios, this is currently not realistic, taking into consideration that this model is one of the first proof-of-concept studies in this anatomical location.

Moreover, when type 3 transition zones are present in the cervix, the most important region to carefully inspect is internal, which makes it difficult for both physicians and deep learning models to accurately identify lesions. This intrinsic anatomical constraint, known beforehand, poses a difficult problem for algorithm development and lesion prediction. Additionally, cervical mucosa is fragile, resulting in the occurrence of minor bleeding, even in the absence of any instrumentation, during colposcopy. Given the black box nature of this technology, we do not know if the presence of the blood can influence the accuracy of the model. Nonetheless, considering the fact that blood can be present in all of the categories mentioned (non-stained, acetic acid- and Lugol-stained, and post-manipulated), and given the high-performance metrics of this CNN, we may infer that the model is not influenced by the presence of blood.

## 5. Conclusions

This AI algorithm demonstrated an effective performance in identifying and distinguishing between LSILs and HSILs, demonstrating the potential for altering patient management. This model was developed using frames from the entire colposcopy examination, encompassing non-stained, acetic acid- and Lugol-stained, and post-manipulated frames. This development can signify an incremental advancement, in comparison to the previous literature, increasing its applicability in a clinical setting. It is important to note that this is an initial, retrospective, and single-center study, relying on a low number of still frames. Acknowledging the necessity for an improved robustness and a broader applicability, our ongoing goal is to significantly expand the dataset for the CNN and explore the inclusion of additional centers.

## Figures and Tables

**Table 1:** Confusion matrix of the test set versus histopathological classification (considered the final diagnosis).

		Final Diagnosis	
		HSIL	LSIL
CNN Classification	HSIL	870	20
	LSIL	3	1377

CNN: Convolutional Neural Network; HSIL: high-grade squamous intraepithelial lesion; LSIL: low-grade squamous intraepithelial lesion.

## Data Availability

The data are available upon reasonable request.
